# Effects of *DSPP* Gene Mutations on Periodontal Tissues

**DOI:** 10.1055/s-0041-1726416

**Published:** 2021-05-24

**Authors:** Zhaojun Jing, Zhibin Chen, Yong Jiang

**Affiliations:** 1 Department of General Dentistry II, Peking University School and Hospital of Stomatology, National Clinical Research Center for Oral Diseases & National Engineering Laboratory for Digital and Material Technology of Stomatology, Beijing, People's Republic of China; 2 Department of Periodontology, Peking University School and Hospital of Stomatology & National Clinical Research Center for Oral Diseases, National Engineering Laboratory for Digital and Material Technology of Stomatology, Beijing Key Laboratory of Digital Stomatology, Beijing, People's Republic of China

**Keywords:** dentin sialophosphoprotein, genetic mutations, periodontal defects

## Abstract

Dentin sialophosphoprotein ( *DSPP* ) gene mutations cause autosomal dominantly inherited diseases. *DSPP* gene mutations lead to abnormal expression of DSPP, resulting in a series of histological, morphological, and clinical abnormalities. A large number of previous studies demonstrated that DSPP is a dentinal-specific protein, and *DSPP* gene mutations lead to dentin dysplasia and dentinogenesis imperfecta. Recent studies have found that DSPP is also expressed in bone, periodontal tissues, and salivary glands. DSPP is involved in the formation of the periodontium as well as tooth structures. DSPP deficient mice present furcation involvement, cementum, and alveolar bone defect. We speculate that similar periodontal damage may occur in patients with *DSPP* mutations. This article reviewed the effects of *DSPP* gene mutations on periodontal status. However, almost all of the research is about animal study, there is no evidence that *DSPP* mutations cause periodontium defects in patients yet. We need to conduct systematic clinical studies on *DSPP* mutation families in the future to elucidate the effect of *DSPP* gene on human periodontium.

## Introduction

The dentin sialophosphoprotein ( *DSPP* ) gene consists of five exons and four introns located on chromosome 4q21. Previously, the DSPP was believed to be expressed only in dentin. 1 To date, 40 pathogenic mutations of the *DSPP* gene have been identified in different ethnic groups, 17 of which are located in the DSP region and 23 in the DPP region. 1 2 3 4 5 6 7 A proposal was made in 1973, based on clinical and imaging characteristics and the expression of *DSPP* gene mutations in dentin, to divide hereditary dentin defects into two distinguishable groups, the dentine dysplasia (DD) and the dentinogenesis imperfecta (DGI), both of which are autosomal dominant genetic diseases. 8 According to the clinical and imaging manifestations, DGI can be further classified into type I, II, and III and DD into type I and type II. 8 9 The most common clinical disease is DGI-II, namely hereditary opalescent dentin. 8 DGI-II, DGI-III, and DD-II are mostly caused by mutations of the same gene, the *DSPP* gene. 1 10

The reason why *DSPP* gene mutations can cause abnormal tooth development is that DSPP is related to the mineralization of hydroxyapatite (HA). The mineralized tissues, such as dentin, cementum, and bone, are developed through similar mechanisms with similar composition to each other. 11 Odontoblasts, cementoblasts, and osteoblasts secrete unmineralized type-I collagen-rich matrices. These unmineralized organic areas are transformed into mineralized phases after the deposition of hydroxyapatite. This dynamic biomineralization process involves active interactions among several molecules, including type-I collagen and numerous noncollagenous proteins (NCPs), of which DSPP is a member of its own category. 11 DSPP is believed to be specifically expressed in dentin in the early stage and has a significant effect on the formation and mineralization of dentin. 8 12 13 It is also expressed in ameloblast cells at a transient level. 14 Subsequently, DSPP is also found in other nondental tissues such as bone, 15 16 periodontal tissues, 17 18 19 20 21 22 kidney, 23 salivary gland, 24 25 26 and mammary gland. 27 *DSPP* gene mutations not only cause hereditary dentine defects, but also affect the formation of other soft and hard tissues. 16 17 24 27 28 29 30 Basing on the critical role of periodontal tissue in oral health, the periodontium defects caused by *DSPP* gene mutations have attracted more and more attention from researchers. Dentine defects caused by *DSPP* gene mutations are usually manifested as crown discoloration, enamel dislodged, dentin attrition, pulp alterations, and periapical lesions. 9 The defective teeth can still function by prosthodontic rehabilitation. However, more serious periodontium defects, especially alveolar bone defects, can lead to teeth loss and a lack of bone mass, making it more difficult to repair. Therefore, we aim to elucidate the association between *DSPP* gene mutations and defects in alveolar bone and other periodontal tissues.

## Clinical Study of Patients with Periodontium Defects Caused by *DSPP* Gene Mutation

A large number of studies have confirmed the existence of periodontal defects in DSPP-deficient mice, but little attention has been directed to the periodontal status of patients with *DSPP* gene mutation. At present, there are few relevant clinical studies and the only study is shown here.

Porntaveetus et al suggested *DSPP* mutation caused alveolar bone damage by studying the characteristics of alveolar bone cells in patients with dentinogenesis imperfecta (DGI) due to *DSPP* mutation. 22 The researchers performed basic research in a family with a *DSPP* heterozygous pathogenic missense mutation presented with defective dentin and periodontium. Heterogeneous cells were isolated from the human alveolar bone of patients with the *DSPP* mutation and compared with those of healthy donors. 22 In comparison, DGI patients with the *DSPP* gene mutation showed reduced alveolar bone cell proliferation and colony formation units, delayed cell spreading, and inhibited osteogenic differentiation as well as altered expression of alkaline phosphatase (ALP), COL1, and OCN. 22 Porntaveetus et al research demonstrated abnormal bone characteristics and altered periodontium features were observed in the patient with DGI caused by the *DSPP* mutation. 22 However, it should be pointed out that the clinical investigation in this study only involved one adult and one child, and the characteristics of alveolar bone cells was analyzed in only one adult. In addition, the details on oral clinical examination like probing depth, clinical attachment loss, bleeding on probing etc. are not shown in this study.

## Three Cleavage Fragments of DSPP and Their Functional Roles

Studies showed that DSPP exists in the extracellular matrix (ECM) as three cleavage fragments: DSP, DPP, and glycosylation dentin sialoprotein (DPG/DSP-PG). 11 21 31 32 DPP and DSP were discovered in 1967 33 and 1981, 34 respectively. The discovery of DSP and DPP in ECM was earlier than DSPP, but their effects were unclear at that time. 33 34 After 13 years later in 1994, it was confirmed that DSP and DPP came from two fragments of the same protein DSPP. 12 35 Later, the third fragment of DSPP, the proteoglycan form of DSP, that is, DPG, was confirmed and purified in 2005. 32

DSP, DPP, and DPG play different roles in the formation and mineralization of mineralized tissue. 17 21 36 37 38 The proteolytic processing of DSPP into DPP, DSP, and DPG is an essential activation step for its biological function in biomineralization. 18 36 In other words, the role of DSPP in biomineralization relies on its cleaved products. Various studies have shown DPP to be an important initiator and modulator of the formation and growth of HA. 37 39 40 DPP is involved in the maturation of mineralized dentin. 37 In addition, DPP has been suggested to regulate the expression of the marker genes of dentin and bone via the SMAD-pathway and integrin/MAPK signaling pathway. 41 42 Although DSP has been shown to regulate the initiation of mineralization, it appears not to have a functional role in the maturation of dentin. 37 In vitro studies have demonstrated that DSP promotes human dental pulp cells to differentiate into odontoblast-like cells. 38 In addition, DSP has been shown in the alveolar bone, cementum, and periodontal ligament (PDL). It suggested that DSP is involved in the formation of the periodontium. 17 N-terminal DSP was shown to promote bone formation by accelerating osteoblast cell proliferation and differentiation and inducing the expression of osteogenic markers, such as COL1, Runx2, Osterix, and ATF. 43 However, little is known about the effects of DPG.

## Animal Study on Periodontal Defects Caused by *DSPP* Gene Mutation/Deletion

Periodontal tissue is the supporting tissue of teeth. It is composed of the gingiva, cementum, PDL, and alveolar bone. 44 As a part of periodontal tissue, the PDL is the bridge connecting tooth and alveolar bone, and plays an important role in tooth support and alveolar bone remodeling. 45

### DSPP is Involved in the Development of Periodontal Tissues

The genomic organization of the *DSPP* gene is very similar among mice, rats, and humans. 46 47 In 2004, Baba et al were among the first group to demonstrate DSP was expressed in the periodontal tissues of rats. 17 In this study, the root formation of molars in rats was systematically studied. The specific anti-DSP polyclonal and monoclonal antibodies were used to detect the DSP, and the high-sensitivity RNA probe was used to detect the DSP mRNA transcripts in situ. The expression of the DSP gene in osteoblasts, odontoblasts, and fibroblasts was confirmed. Although the gene expression level of osteoblasts was low, the staining intensity in the alveolar bone matrix was similar to that in dentin. These results suggest that DSP plays a vital role in the formation of the periodontium, especially alveolar bone. 17

The PDL contains PDL stem cells (PDLSC) that have a high proliferative capacity and could differentiate into different cell lineages, such as osteoblast-like cells, cementoblast-like cells, and fibroblast-like cells. Furthermore, PDLSCs are extremely important for maintaining PDL stability, repairing cementum tissue damage, and achieving functional cell renewal. 48 49 In 2013, Ozer et al studied the expression pattern and distribution of DSP fragments in mouse periodontium at the transcriptional and translational levels using in situ hybridization and immunohistochemical analyses, and confirmed that DSP was expressed in PDL and alveolar bone at various stages of root development. 20 The recombinant COOH-terminated DSP fragment (rC-DSP) induces cell proliferation and differentiation by promoting the expression of tooth/bone-related markers, transcription factors, and growth factors, as well as by the formation of mineralized tissue and ALP activity. DSP was proved for the first time to be able to promote the adhesion, migration, proliferation, and differentiation of PDLSC and PDL cells, thus promoting the formation, repair, and regeneration of periodontal tissue. 20

### The Loss of DSPP Expression or the Overexpression of the NH2 Terminal Expression Products of DSPP Caused Periodontium Defects

Gibson et al conducted a series of studies on DSPP. In addition to proving the important role and mechanism of DSPP in the process of dentin formation, 30 36 they also found that DSPP was closely related to the formation and health maintenance of periodontal tissue. 18 19 21 This paper mainly discusses the close relationship between DSPP and periodontal tissue. In 2013, Gibson et al conducted a study on *DSPP* gene knockout mice, which confirmed that DSPP deletion would cause severe damage to the periodontium, and suggested that DSPP plays a key role in maintaining the structural integrity of periodontium. 19 X-ray plain film and micro-CT were used to evaluate the morphology and structure of the whole mandible of *DSPP* gene knockout mice, which showed that alveolar bone defects were more serious than in normal mice, especially in the furcation area of mandibular molars. Histochemical observation revealed alveolar bone loss, inflammatory cell infiltration, detachment of the PDL, and the apical migration of epithelial attachment, suggesting the occurrence and progression of periodontitis. 19 Scanning electron microscope data further confirmed alveolar bone loss and showed a significant loss of cementum. The mineral content around osteoblasts in DSPP deficient mice was significantly reduced, which was manifested as alveolar bone and cementum defects, secondary damage to the PDL located between alveolar bone and cementum. 19 These structural changes lead to bacterial infiltration, periodontal inflammation, further loss of alveolar bone and periodontal pocket formation, periodontal diseases occurred in the end. Gibson et al pointed out in another paper in 2013 that proteolysis of DSPP was a necessary condition for the formation and maintenance of a healthy periodontium. 18 The results showed that transgenic mice expressing the uncleavable full-length DSPP in the *DSPP* knockout ( *DSPP* -KO) background (named *DSPP* -KO/D452A-Tg mice) had severe alveolar bone loss and obvious inflammatory cell infiltration in the furcation area of mandibular first molars. However, the mice expressing the normal *DSPP* transgene in the *DSPP* -KO background (designated *DSPP* -KO/normal-Tg mice) could reverse alveolar bone damage, show epithelial attachment at the cemento-enamel junction, and promote the deposition of healthy alveolar bone. The study suggested that DSPP proteolysis was significantly associated with periodontal tissue formation and health maintenance. 18 Gibson also suggested that the periodontal defects observed in DSPP-deficient mice are due to intrinsic defects in alveolar bone and cellular cementum due to DSPP dysfunction but are not disease secondary to chronic periodontitis. It is likely the alveolar bone loss occurs earlier than chronic periodontitis, and periodontitis can exacerbate the periodontal defects in DSPP-deficient mice as chronic periodontitis itself could also cause alveolar bone loss and PDL damage. 18 Gibson et al mentioned it remained to be seen whether treating and preventing chronic periodontitis could slow or save periodontal defects in DSPP-deficient mice. In 2014, the Gibson et al made further research on the NH2 terminal expression products of DSPP, the DSP and DPG. Compared with *DSPP* knockout mice, they concluded the transgenic mice overexpressing the NH2-terminal fragment of DSPP in the *DSPP* knockout background (named as “ *DSPP* KO/DSP Tg” mice) showed more severe defects in the alveolar bone and cementum with remarkably altered canalicular systems around the osteocytes, and more apical migration of the epithelial attachment with severe inflammation in molar furcation region. 21 Overexpression of NH2 end of DSPP can also aggravate the destruction of periodontal tissue, suggesting NH2 end fragment of DSPP protein may inhibit the formation and mineralization of the alveolar bone and cementum. 21

Shi et al study also supported that DSPP deficiency could lead to periodontal tissue defects. In addition, Shi et al focused specifically on the periodontal phenotype of *DSPP* heterozygotes and their age-dependent periodontal damage. 50 The study divided mice into three groups; *DSPP* genotype of wild-type group ( *DSPP* +/+ ), heterozygous HET group ( *DSPP* +/− ), and knockout KO group ( *DSPP* −/− ). The result showed, except for similar dental phenotypes to DD-II in *DSPP* heterozygous group, the periodontium was also affected; exhibiting apical migration of the junctional epithelium, decreased alveolar bone height and width, and inflammatory cell infiltration leading to periodontal tissue destruction. Both the dental and periodontal phenotypes are age dependent and more severe at 18 months than at 12 months. The study showed that *DSPP* heterozygous mice developed severe periodontitis with age. 50

## The Relationship between Plaque Microorganism and Alveolar Bone Loss

From the above study, we learnt the mechanisms of the alveolar bone defects caused by *DSPP* gene mutations include primarily the inhibition of osteoblastic differentiation of osteocytes, the reduction of bone formation, and the subsequent inflammatory infiltration leading to continuous alveolar bone destruction. Alveolar bone loss is mainly due to periodontal inflammation clinically. The etiology of periodontitis is multifactorial, among which dental plaque is the initiating factor. 51 52 In particular, a group of specific gram-negative anerobic species known as the “red complex” results in chronic inflammation. 53 The subgingival plaque biofilm causes inflammation and elicits immune responses of the host, leading to irreversible destruction of the periodontal tissues eventually, especially alveolar bone and PDL of the susceptible host. 51 54 55

## Summary and Outlook

In summary, *DSPP* gene mutations cannot only lead to typical hereditary dentin defects but also lead to defects of the periodontal tissue. Therefore, we need to update our understanding of the effects of *DSPP* gene mutations on oral health. The mechanisms for the severe periodontium defects and even teeth loss caused by *DSPP* gene mutations can be summarized as the following two aspects. On the one hand, the deficiency of DSPP, caused by mutations in the *DSPP* gene, leads to the failure of the normal formation and mineralization process of periodontal tissues, which is manifested as alveolar bone defect and the apical migration of the junctional epithelium. On the other hand, congenital PDL defects make this area a vulnerable point for the colonization and reproduction of oral microorganisms, leading to infiltration of inflammatory cells and creating suitable conditions for initiating development of periodontitis. When the periodontium of the *DSPP* gene mutations is doubly suppressed by genetic defects and microecology, the periodontal prognosis of the *DSPP* gene mutations is not optimistic. Whether the two have superimposed effects still needs to be further elucidated. However, almost all of the studies on the effect of DSPP on periodontal tissue formation are from animal studies, and there is no exact evidence that patients with DSPP gene defects have severe periodontium defects yet. More clinical studies are therefore needed in this field.

In addition, it has been reported that periodontal inflammation in *DSPP* mutant mice is age-dependent. We speculate periodontal defects in patients with *DSPP* mutations may also worsen with age. Again, this is only an animal study, and no relevant clinical studies were conducted. Because the health of periodontium is critical to tooth survival, the effect of *DSPP* mutations on periodontal tissue deserves our attention. Although the current research on *DSPP* gene mutations is mainly confined to animal experiments, the conclusions of these studies help us to clarify the direction of future research. In the future, we need to conduct systematic clinical studies on *DSPP* mutation families. Two questions remain to be answered through further in vivo studies. First, whether *DSPP* mutation causes periodontal defects in patients, even age-dependent periodontal defects? Second, whether *DSPP* gene mutation is associated with periodontitis?
